# MPFL Reconstruction in Skeletally Immature Patients: Comparison Between Anatomic and Non-Anatomic Femoral Fixation—Systematic Review

**DOI:** 10.3390/children11111275

**Published:** 2024-10-22

**Authors:** Georgios Kalinterakis, Iakovos Vlastos, Elina Gianzina, Savvas Dimitriadis, Konstantinos Mastrantonakis, Efstathios Chronopoulos, Christos K. Yiannakopoulos

**Affiliations:** 1 School of Physical Education and Sport Science, National and Kapodistrian University of Athens, 17237 Athens, Greece; captaindot@phed.uoa.gr (I.V.); elgianzi@phed.uoa.gr (E.G.); savvasdim@phed.uoa.gr (S.D.); kmastra@med.uoa.gr (K.M.); c.yiannakopoulos@phed.uoa.gr (C.K.Y.); 2 Laboratory for Research of the Musculoskeletal System “Th. Garofalidis”, Medical School, National and Kapodistrian University of Athens, KAT General Hospital, Kifissia, 14561 Athens, Greece; echronop@med.uoa.gr

**Keywords:** MPFL, reconstruction, open physis, distal femoral fixation

## Abstract

Background: MPFL reconstruction in children with open physis may be challenging, as a major concern during the surgery is to preserve the distal femoral physis. The purpose of this study was to compare the complication rate and the patient-reported outcomes in skeletally immature patients who underwent MPFL reconstruction using an anatomic (A) or non- anatomic (NA) surgical technique. Methods: For this systematic review, the authors adhered to the PRISMA guidelines. The literature search was conducted from inception to 31 May 2024. Three databases were used: Pubmed, Scopus and Cochrane library. We included skeletally immature patients who underwent MPFL reconstruction for chronic or recurrent patellar instability. The included studies should describe the surgical technique, report clinical outcomes and complications. Patients with closed physis, prior ipsilateral knee surgery, concomitant surgical procedures except for lateral retinacular release, multiligament knee injury, congenital or acute patellofemoral instability, hyperlaxity or less than 12 months follow up were excluded. Risk of bias was assessed using ROBINS-I, MINORS and MCMS scores. Results: Data from 304 procedures were collected, of which 208 were performed using an anatomic technique and 96 using a non-anatomic technique. Patient age at the time of surgery ranged from 8 to 17 years. The follow-up time ranged between 12 and 116.4 months. Postoperative Kujala (−0.73, *p* = 0.55) and Tegner (−0.70, *p* = 0.80) scores were better in the anatomic group compared to the non-anatomic one. Higher rates of recurrent instability (OR 0.91; 95%CI 0.44–1.86, *p* = 0.85), redislocation (OR 1.21; 95%CI 0.42–3.51, *p* = 0.8), subluxation (OR 0.73; 95%CI 0.29–1.83, *p* = 0.62), a positive apprehension test (OR 0.92; 95%CI 0.27–3.13, *p* = 0.89), stiffness (decreased ROM) (OR 1.63; 95%CI 0.33–1.72, *p* = 0.54) and reoperation (OR 1.16; 95%CI 0.35–3.80, *p* = 0.8) were reported in papers using the anatomic technique. Conclusions: The findings of this systematic review reveal that there is no significant difference between anatomic and non-anatomic MPFL reconstruction techniques in terms of patient-reported outcomes and complications. Thus, the choice of surgical technique might be left up to surgeon’s preference. Further high-quality, pediatric-oriented studies with long-term follow–up are needed to better guide clinical decision-making.

## 1. Introduction

Recurrent lateral patella dislocation is a common injury in skeletally immature patients, with an annual incidence of 29 per 100,000 among individuals aged 10 to 17, and the peak occurrence of first-time dislocation at age 15 [1,2,3,4]. In patients under the age of 14, the recurrence rate can reach up to 60%, highlighting the elevated risk in young, active individuals [5]. The medial patellofemoral ligament (MPFL) is essential as one of the main stabilizers of the patella, providing 50–60% of the medial restraining force against lateral displacement, especially during the first 30° of knee flexion [6]. MPFL reconstruction has gained significant popularity in the past decade, becoming the leading surgical technique for stabilizing recurrent patellar instability, replacing less anatomic procedures [7]. A major concern during MPFL reconstruction in adolescents is to preserve the distal femoral physis. For this reason, non-anatomic reconstruction techniques have been employed at first, which use the femoral insertion points of the medial collateral ligament (MCL) or the adductor magnus tendon as reference markers for placing the MPFL’s femoral insertion. These anatomic structures act as a pulley for the graft and, thereby, the creation of a bone tunnel, which can damage the distal femoral physis, is avoided. However, in the last decade, several authors have highlighted the significance of anatomic repairs, introducing physeal-sparing anatomic reconstruction techniques with promising results [8,9,10]. Anatomic MPFL reconstruction is considered as that which is distal to the physis. The anatomic area corresponds to radiographic landmarks, as established by Schottle and Redfern [11,12].

The objective of this study was to investigate which surgical technique is more favorable in pediatric patients with open physis who underwent medial patellofemoral ligament (MPFL) reconstruction, whether anatomic (A) or non-anatomic (NA). We focused on patient-reported outcomes and the complications.

## 2. Materials and Methods

### 2.1. Study Selection

According to the Declaration of Helsinki, the study received approval from our Institutional Review Board (approval number 1652-12/06/2024) and adhered to the 2020 PRISMA guidelines. The PICO framework was applied as follows:

- P (Population/Pathology): patellar instability/skeletally immature patients;
- I (Intervention): MPFL reconstruction;
- C (Comparison): anatomic versus non-anatomic technique;
- O(Outcomes): patient-reported outcome measures (PROMs), return to sport rates and complication rates;
- S (Studies): human studies with >12 months follow-up and involving >10 patients.

**Figure 1:**
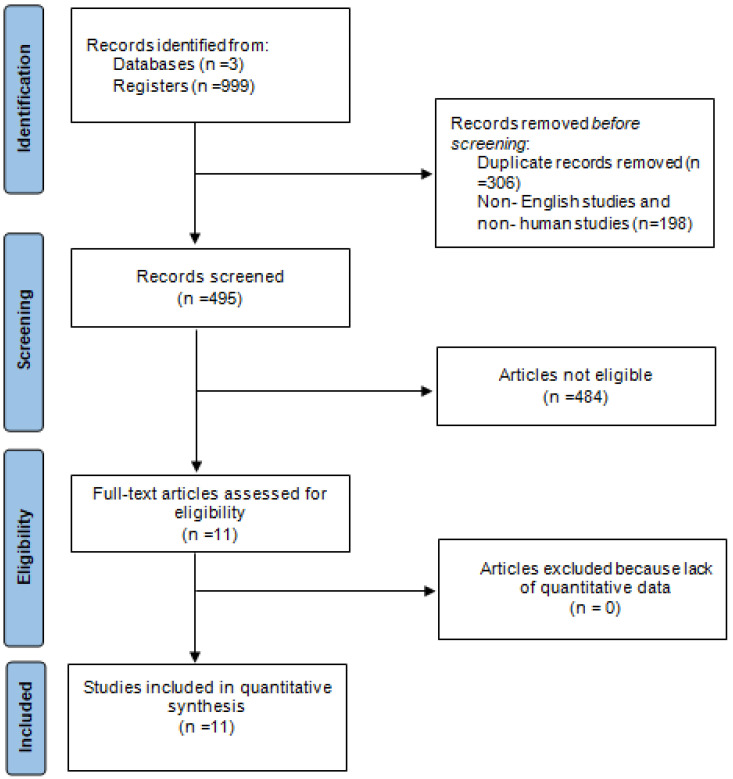
Search strategy flow chart.

A comprehensive literature search was conducted using PubMed, Cochrane Library and Scopus databases (Figure 1). The search was performed from inception to 31 May 2024. The search keywords employed were as follows: patellofemoral, dislocation or instability, open physis or adolescent or skeletally immature, MPFL or medial patellofemoral ligament, surgery or reconstruction.

Through this search, all related studies were identified. Moreover, the bibliographies of all included studies were reviewed to identify any further relevant studies. This review protocol was registered on the International Prospective Register of Systematic Reviews.

### 2.2. Inclusion and Exclusion Criteria

This systematic review includes studies that involved skeletally immature patients who underwent MPFL reconstruction for chronic or recurrent patellar instability using either an anatomic or non-anatomic technique. To be included, studies had to describe the surgical technique and report clinical outcomes and complications. The exclusion criteria during screening were as follows:

- Patients with a closed physis;
- Animal studies;
- Cadaveric studies;
- Patients with prior ipsilateral knee surgery;
- Studies involving patients with congenital or acute patellofemoral instability;
- Studies focusing solely on patients with hyperlaxity or multiligament injury;
- Level of evidence V studies and reviews;
- Non-English studies;
- Studies with fewer than 12 months of clinical follow-up;
- Case series involving fewer than 10 patients;
- Concomitant surgical procedures other than lateral retinacular release.

In studies that included patients with both open and closed physis, we only included those that provided sufficient detail on the endpoints of interest to allow stratification based on skeletal maturity.

### 2.3. Assessment of Methodological Quality

For the methodology quality assessment, the Modified Coleman Methodology Score (MCMS) was used. This tool operates on a scale from 0 to 100. Scores of 85 to 100 were classified as excellent; 70 to 84, as good; 55 to 69, as fair; and <55, as poor. The MCMS was developed to evaluate outcomes related to surgery for patellar tendinopathy, making it suitable for assessing patellar stabilization procedures [13]. The risk of bias in the included studies was assessed using the Methodological Index for Nonrandomized Studies (MINORS) score and the Risk Of Bias In Non-randomized Studies of Interventions (ROBINS-I) tool. MINORS is a validated scoring system with 12 items for comparative studies and 8 items for case series. It evaluates various aspects, such as the study’s objective, patient inclusion criteria, data collection, endpoints, follow-up, rate of loss during follow-up, sample size calculation, the inclusion of a control group, group equivalence and the adequacy of statistical analysis. It has a potential range of 0 to 16 for non-comparative studies and 0 to 24 for comparative studies [14]. ROBINS-I tool rates studies as “Low”, “Moderate”, “Serious” and “Critical” risk of bias by analyzing 7 domains: confounding bias, selection bias, bias in classification of interventions, deviation bias, missing data bias, measurement bias and reporting bias [15]. Two investigators, G.K. and K.M., calculated the scores, resolving any disagreements through discussion and consensus. The results of these evaluations are shown in Table 1.

### 2.4. Data Collection and Analysis

Two authors (G.K. and K.M.) independently assessed the data. The process of extracting data began with the organized documentation of the information from the studies being analyzed. This entailed constructing a detailed table in which various important elements were carefully recorded. Disagreements between the authors were discussed and resolved collaboratively. Particular emphasis was given to the method of femoral graft fixation in each surgical technique. Anatomic techniques were described as those employing the radiographic signs identified by Schottle [12]. In contrast, non-anatomic techniques utilized the femoral insertion of either the medial collateral ligament (MCL) or the tubercle of the adductor magnus tendon as reference landmarks for MPFL insertion [26]. Information collected included authorship, year of publication, the study’s level of evidence, number of patients, mean age of the sample at the time of surgery, number of procedures and mean follow-up duration. Patient-reported outcome measures (PROMs) at baseline and final follow-up were the Kujala score [27] and the Tegner Activity Scale score [28]. The following complications were assessed: recurrent patella instability, further re-dislocations or subluxations, positive apprehension tests, patella fractures, and revision surgeries.

Numerical demographic data are summarized using descriptive statistics, while categorical variables (e.g., patellar fractures, redislocations) are expressed as percentages. Because the available data were heterogeneous and of low quality, a subjective synthesis was performed, and the range of outcome measure values is reported. Binary data were analyzed using odds ratios (OR) with corresponding confidence intervals (CI). All analyses were conducted using SPSS version 29.0 (IBM Corporation, Somers, New York, NY, USA).

## 3. Results

### 3.1. Search Results

The literature search yielded a total of 999 papers: 655 articles from PubMed, 303 articles from Scopus and 41 articles from the Cochrane Library. After the removal of duplicate studies, 693 studies remained. Non-English studies with unavailable full texts and non-human studies were excluded, reducing the number to 495 articles. A manual review of the abstracts was then conducted, and 484 articles were excluded for the following reasons: not being relevant to the study’s purpose (n = 415), study type and design (n = 25), small sample size (n = 4), acute patellofemoral instability and severe dysplasia (n = 8) and skeletally mature patients (n = 32). Full texts of the remaining articles were retrieved and assessed for inclusion. Finally, 11 studies fulfilled the criteria for our review (Figure 1).

### 3.2. Quality of the Included Studies

The MCMS scores for the 11 included studies are summarized in Table 1. The average MCMS score was 57.45, ranging from 48 to 68. None of the studies achieved an excellent or good rating, with six studies receiving a fair rating and five classified as poor. Overall, the quality of the studies was fair. The results of MINORS score and ROBINS-I tool are also presented in Table 1. The average MINORS score was 9.45, with scores ranging from 7 to 12. In terms of ROBINS-I tool, all 12 studies showed a moderate risk of bias due to confounding, as there were no prognostic variables that predicted baseline intervention. All the studies included participants who were eligible for the target trial (low risk of bias). Bias in classification of interventions was low in all studies as intervention status was well defined. No studies deviated from the intended intervention (low risk of bias). Overall, 10 studies did not show bias due to missing data (low risk of bias), and in two studies, only 80–90% completed the final follow-up (moderate risk of bias). The vast majority of the studies had a retrospective nature (10 out of 12), so in this case, the risk of bias was high. Finally, bias due to selective reporting was not observed in any of the studies (low risk of bias). A detailed description of the ROBINS-I tool can be found in the supplemental section.

### 3.3. Patient Demographic

This systematic review includes 288 patients with open physis who underwent MPFL reconstruction between 2008 and 2020. The patients’ ages at the time of surgery ranged from 8 to 17 years. The follow-up period varied between 12 and 116.4 months. Overall, 304 MPFL reconstructions were included, with 208 performed using an anatomic technique and 96 using a non-anatomic technique. Only 20% of the knees (69 out of 304) had severe trochlea dysplasia (Dejour type C and D), 25 in the non-anatomic group and 44 in the anatomic group, respectively. The patella height was evaluated with the Insall–Salvati (IS) and the Caton–Deschamps (CD) ratio. The mean IS and CD scores were 1.24. The mean tibial tubercle–trochlear groove distance (TT-TG) was 1.56 (1.51 for the anatomic group and 1.65 for the non-anatomic group). The characteristics of each study are presented in Table 2 and Table 3.

### 3.4. Clinical Outcomes

All studies, except two [20,21], provided postoperative data using the most commonly used Kujala score, and nearly half of them (5) did not state any pre-operative data [19,20,21,22,23]. In the anatomic reconstruction group, the mean postoperative Kujala scores ranged from 80.3 to 97.9, while in the non-anatomic reconstruction group, the mean postoperative Kujala scores ranged from 71 to 100. The Tegner score was reported in fewer studies. Specifically, the mean Tegner scores were evaluated in four studies using the anatomic technique [8,18,22,25] and in one study using the non-anatomic technique [17]. The mean postoperative score ranged between 3 and 6.3. There were no statistically significant differences in Kujala or Tegner scores between the two techniques, either preoperatively or postoperatively, as depicted in Table 4.

### 3.5. Complications

Table 5 summarizes the complications of each study. In the anatomic group, the majority of the studies used an interference screw for graft fixation in the distal femoral epiphysis [8,18,19,20,22]. Notably, no hardware-related complications were reported in any of the included studies.

No complications were reported in three studies, two of them using a non-anatomic and one the anatomic technique. Recurrent instability was the most frequently reported complication and defined as recurrent dislocation or subluxation events, as reported by the patient. Higher rates of recurrent instability (OR 0.91; 95%CI 0.44–1.86, *p* = 0.85), redislocation (OR 1.21; 95%CI 0.42–3.51, *p* = 0.8), subluxation (OR 0.73; 95%CI 0.29–1.83, *p* = 0.62), stiffness (decreased ROM) (OR 1.63; 95%CI 0.33–1.72, *p* = 0.54) and reoperation (OR 1.16; 95%CI 0.35–3.80, *p* = 0.8) were reported in papers using the anatomic technique. Additionally, more patients in the anatomic technique group stated that they had experienced a positive apprehension test compared to the non-anatomic one (OR 0.92; 95%CI 0.27–3.13, *p* = 0.89).

Overall, a total of 74 (24.3%) complications were reported: 18 (5.9%) redislocations, 21(6.9%) subluxations, 14 (4.6%) reoperations, 12 patients (3.9%) with positive apprehension tests and 9 patients (3%) with postoperative stiffness. Three studies mentioned no relationship between high trochlea dysplasia (Dejour type C and D) and postoperative complications [16,17,22] and one reported more complications in patients without bone abnormality [19].

### 3.6. Return to Sport

The mean time to return to sport was 5.85 ± 1.01 months in the anatomic technique group. Regarding the non-anatomic technique group, none of the included studies reported this outcome measure. For those who did not return to sport (9.8%), the most common causes were the fear for re-injury and lack of interest. Approximately, 90% of the patients returned to sports at a higher or at least at the same level than pre-operatively.

## 4. Discussion

In this systematic review, the outcomes and complications of MPFL reconstruction in skeletally immature patients was examined. Anatomic and non-anatomic MPFL reconstruction techniques did not show statistically significant differences in all examined variables. The included studies were of low-quality and showed substantial heterogeneity. This may be considered a paradox given the relatively high rate of patella dislocation in adolescent patients.

Over the last two decades, the interest around MPFL reconstruction has shown a significant increase [29]. Compared to adults, surgical treatment in young patients involves significant and technically demanding considerations. Proper anatomical placement of femoral and patellar tunnels is essential for accurately reconstructing the MPFL anatomy. Technically, correct femoral graft placement is critical for a successful outcome. In skeletally immature patients, bone tunnel drilling, particularly in the femur, is avoided to reduce the risk of injuring the distal femoral physis. Instead, the femoral attachment of the MPFL is guided by the insertion points of either the posterior portion of the medial collateral ligament (MCL) or the adductor tendon. These anatomic structures are used as a pulley where the graft can be looped. So, the latter allows a more “dynamic” soft tissue fixation rather than a stable bone fixation. However, these “sling procedures” are not considered anatomic. Lind et al. [16], in their cohort, reported a troubling revision rate of 21% using a non–anatomic MPFL reconstruction technique. Another similar study reported good clinical outcomes with a redislocation rate of 2.85% [17], while two other authors did not mention any complications at all [23,24]. In our study, the total recurrence rate in the non-anatomic MPFL reconstruction group was 4.2%, including 1.6% dislocations and 2.6% subluxations. These findings are in accordance with previous reviews, which found a recurrence rate ranging from 5% to 7% [30,31].

MPFL reconstruction is considered a more anatomic procedure and, thus, bony fixation that preserves the femoral origin of the ligament is advocated [16,18]. Although most current evidence indicates that MPFL’s femoral insertion point in pediatric patients is located at or just below the physis, this topic still sparks controversy [22]. Various studies have shown that the average proximity of the Schottle point to the physis is between 3.2 and 8.5 mm [32,33,34]. Intraoperative X-ray guidance is essential to prevent physeal injury and to ensure proper tunnel placement. In adults, a perfect lateral radiograph suffices to establishing the femoral insertion point [35]. In skeletally immature patients, due to the fact that the distal femoral physis is concave in some parts, identification based only on the lateral X-ray might be fallacious. Thus, the surgeon should firstly pinpoint Schottle’s point on the lateral radiograph and then verify on the anteroposterior radiograph that the point of interest is located distal to physis [36,37,38,39]. Apart from that, Nguyen et al. pointed out that the trajectory of the tunnel also plays an important role in minimizing possible damage to the physis and they recommend distal and posterior angulation of 15 to 20 degrees [40]. Notably, the results of our systematic review did not reveal any growth plate injury or growth disturbance in the included studies that used the anatomic technique.

Regarding the patient-reported outcome measures (PROMs), both techniques were associated with significant improvement, without a statistically significant difference. Although most of the studies used the Kujala score, there was no consistency and standardization in PROMs reporting. Apart from this, in pediatric clinical settings, patient-centered care includes family caregivers, as the medical team usually directs questions to them. These caregivers act as the primary agents in providing care for the patient. However, PROMs completed by caregivers do not genuinely represent “patient-reported” outcomes, as they involve caregivers or healthcare providers reporting on the child’s experiences. Furthermore, the evidence indicating agreement between self-reported outcomes from pediatric patients and proxy-reported outcomes from caregivers is weak [40]. It is essential that future studies focus on developing pediatric-specific PROMs. A recent systematic review also stated that there is a need for more pediatric-specific outcome studies regarding operatively managed traumatic patellofemoral instability for safer interpretation of the results [41]. In our review, no study reported age-specific treatment outcomes.

Recent systematic reviews have shown that MPFL reconstruction for addressing patellar instability in young patients is a reliable and safe procedure, with only minor complications [30,42]. The main outcome measure in our study was recurrent patella instability, with a rate of 12.8%. The latter is in agreement with previous papers, which stated an overall recurrent instability rate from 5% to 13.8% [30,31,43,44]. No significant statistical differences were observed between the compared techniques regarding recurrent instability, as well as non-stability-related complications. Hence, our results did not confirm the current belief that the strictly anatomic technique when it comes to femoral fixation is more successful than the non-anatomic technique [8,16,18,45]. From this perspective, the selection of a specific technique for a given patient is at the discretion of the attending physician.

This systematic review has several limitations that should be acknowledged. Firstly, most of the included studies were retrospective case series, offering a low level of evidence with a lack of randomization and blinding; only four studies incorporated a control group [16,21,24,25]. Additionally, no studies in the literature specifically compare the anatomic and non-anatomic techniques regarding femoral fixation. The number of patients in the anatomic group was significantly higher than those in the non-anatomic group. Furthermore, the analysis did not consider variations in the type and location of patellar fixation. Another limitation of this review is the inconsistency in demographic data presentation and, more critically, in the reporting of complications. The lack of a consistent definition for postoperative complications across the included studies affects the reliability of the reported complication rates. What is more, only one study [20] utilized full-length lower limb X-rays to evaluate leg lengths and coronal angular alignment, which is essential for detecting potential growth arrest that could lead to limb-length discrepancies or angular deformities. Moreover, the patient-reported outcome measures (PROMs) used in the studies are not specifically validated for pediatric populations, potentially impacting the accuracy and relevance of the outcomes. What is more, since most studies reported only mid-term follow-up, there is a possibility that some cases of physeal arrest might be underreported.

Lastly, publication bias should be taken into account. This may favor studies with positive or statistically significant results, distorting the conclusions. That being said, statistical significance does not always imply clinical importance. Small sample sizes and large variability could make it harder to detect an effect, even if one exists. As a result, if there were larger sample sizes, a difference between the two techniques would be easier to be found. From this perspective, modern physicians must not base their practice only on one study, but take into account the broader context of the evidence for their daily surgical practice. For the aforementioned reasons, we cannot indicate which technique is more favorable. Decisions should be made according to surgeon’s skills and patient characteristics. For instance, it would be more prudent to choose the anatomic technique in children near to skeletal maturity. Moreover, anatomic reconstruction may not be as dangerous and risky a procedure as initially thought given that none of the included studies revealed any growth plate disturbance. Another deduction is that “sling procedures“ are safe and effective options, even if they do not follow strictly anatomic rules. In order to confirm the current trend for a more anatomic reconstruction, we need randomized controlled trials with long-term follow-up comparing the two techniques.

## 5. Conclusions

The findings of this systematic review indicate that there is no significant difference between anatomic and non-anatomic MPFL reconstruction techniques concerning patient-reported outcomes and complication rates. Thus, for the time being, the choice of surgical technique might be left up to surgeon’s preference. To identify the optimal clinical decision-making, further high-quality, pediatric-oriented research is needed. Specifically, randomized controlled trials with long-term follow-up should be the gold standard for demonstrating any potential superiority of one technique over the other.

## Figures and Tables

**Table 1:** Results of MINORS, Modified Coleman Methodology score and ROBINS-I tool.

	Authors	MINORS Score Average	MCMS Score Average	ROBINS-I Tool (Overall)	Level of Evidence	Type of Study
1	Nelitz et al., 2012 [8]	12	63	Serious risk of bias	IV	Case series
2	Lind et al., 2014 [16]	9	56	Serious risk of bias	III	Case-control study
3	Machado et al., 2017 [17]	10	68	Moderate risk of bias	III	Prospective cohort study
4	Nelitz et al., 2017 [18]	10	66	Moderate risk of bias	III	Prospective cohort study
5	Pesenti et al., 2017 [19]	7	48	Serious risk of bias	IV	Case series
6	Uppstrom et al., 2019 [20]	8	51	Serious risk of bias	IV	Case series
7	Quinlan et al., 2021 [21]	8	52	Serious risk of bias	III	Case-control study
8	Schlumberger et al., 2021 [22]	11	65	Serious risk of bias	IV	Case series
9	Wang et al., 2023 [23]	10	52	Serious risk of bias	IV	Retrospective cohort study
10	Zhang et al., 2023 [24]	8	51	Serious risk of bias	IV	Case series
11	Leite et al., 2023 [25]	11	60	Serious risk of bias	IV	Retrospective cohort study
	Average Score	9.45	57.45			

**Table 2:** This summary reflects the general characteristics and outcomes reported in the studies included in the review.

	Authors, Year	Patients	Knees (n)	Meanage (Range, Years)	Mean Follow-Up (Range, Months)	Type of Graft	Technique	Return to Sport (Months)	Complications, Number of Cases
1	Nelitz et al., 2012 [8]	21 (15 M/6 W)	21	12.2 (10.3–13.9)	33.6 (24–43.2)	Gracilis	Anatomic	5.3 (4–12)	Stiffness: 1
2	Lind et al., 2104 [16]	20 (9 M/11 F)	24	12.5	39 (17–72)	Gracilis	Non-anatomic	Not reported	Redislocation: 4 Subluxation: 5 Stiffness: 2 Reoperation: 4 Apprehension: 1
3	Machado et al., 2017 [17]	35 (11 M/24 W)	35	15.9 (14–17)	116.4	Gracilis	Non-anatomic	Not reported	Redislocation: 1 Subluxation: 3 Superficial Infection: 1 Anterior knee pain: 5 Donor site pain: 3 Apprehension: 3
4	Nelitz et al., 2017 [18]	25 (9 M/16 F)	25	12.8 (9.5–14.7)	31.2 (24–40.8)	Quadriceps	Anatomic	4.8 (3–11)	No complications
5	Peseni et al., 2017 [19]	25 (19 M/6 W)	27	13.8 ± 2.5	41.1 ± 13.5	Gracilis (19)/ Semitendinosus (8)	Anatomic	7.1 ± 3.5	Redislocation: 1 Wound complications: 5
6	Uppstrom et al., 2019 [20]	49 (30 M/19 W)	54	13.3 ± 1.6	26.4 (12–68.4)	Gracilis/ Semitendinosus	Anatomic	Not reported	Redislocation: 5 Reoperation: 4
7	Quinlan et al., 2021 [21]	16 (9 M/7 F)	17	13.5 ± 1.0	49.2 ± 19.2	Allograft	Anatomic	Not reported	Redislocation: 2 Subluxation: 4 Reoperation: 3 Stiffness: 5
8	Schlumberger et al., 2021 [22]	41 (33 M/8 F)	45	13.8 ± 1.1	51.6 ± 20.4	Gracilis	Anatomic	6.2 ± 3.0	Redislocation: 3 Subluxation: 1 Stiffness: 1 Reoperation: 3
9	Wang et al., 2023 [23]	16 (8 M/8 W)	16	11.56 ± 1.15	25.81 ± 1.42	Adductor magnus tendon	Non-anatomic	Not reported	No complications
10	Zhang et al., 2023 [24]	21 (9 M/12 F)	21	10.7 (8–13)	24–42	Peroneus longus	Non-anatomic	Not reported	No complications
11	Leite et al., 2023 [25]	19	19	14 (11–17)	69.6 ± 20.4	Gracilis	Anatomic	Not reported	Redislocation: 5 Apprehension: 8 Subluxation: 8

**Table 3:** Radiological characteristics of the patients of the included studies. TT-TG: tibial tuberosity–trochlear groove distance, N/A: not available, M: mean, SD: standard deviation.

Radiological Parameters	Total	Anatomic Group	Non-Anatomic Group	*p*-Value
Severe trochlea dysplasia (Dejour C/D)-n%	69	44	25	0.344 ^a^
TT-TG, M ± SD	1.56 (0.43)	1.51 (0.21)	1.65 (0.73)	0.507 ^b^
Caton–Deschamps ratio, M ± SD	1.24 (0.09)	1.28 (0.02)	1.10	0.157 ^b^
Insall–Salvati ratio, M ± SD	1.24 (0.08)	1.24 (0.08)	N/A	-

^a^ Chi-square test, ^b^ Mann–Whitney test, *p* significant at <0.05.

**Table 4:** Analysis of scores according to the employed technique. M: mean, SD: standard deviation.

	Anatomic Technique	Non-Anatomic Technique	*z*-Value	*p*-Value ^a^
	M (SD)	M (SD)		
Kujala Preoperatively	62.66 (10.40)	47 (10.40)	−1.09	0.40
Kujala Postoperatively	91.06 (6.85)	85.31 (13.19)	−0.73	0.55
Tegner Preoperatively	4 (2)	7 (2)	−1.34	0.50
Tegner Postoperatively	5.02 (1.45)	4 (3)	−0.70	0.80

^a^ *p* significant at <0.05 (Mann–Whitney test).

**Table 5:** Analysis of complications according to the employed technique.

Complications	Total (n_1+2_/N_1+2_)	Anatomic Group (n_1_/N_1_)	Non-Anatomic Group (n_2_/N_2_)	*p*-Value ^a^
Recurrent instability	39/304	26/208	13/96	0.85
Redislocation	18/304	13/208	5/96	0.8
Subluxation	21/304	13/208	8/96	0.62
Positive Apprehension test	12/304	8/208	2/96	0.89
Stiffness	9/304	7/208	2/96	0.54
Reoperation	14/304	10/208	4/96	0.8

^a^ Chi-square test, *p* significant at <0.05, n_1_ = number of cases in the anatomic group, N_1_ = total number of patients in the anatomic group, n_2_ = number of cases in the non-anatomic group, N_2_ = total number of patients in the non-anatomic group.

## Data Availability

Data are contained within the article.

## Supplementary Materials

The following supporting information can be downloaded at: https://www.mdpi.com/article/10.3390/children11111275/s1, Figure S1: A detailed description of the ROBINS-I tool.
