# Supplementary material for: MPFL Reconstruction in Skeletally Immature Patients: Comparison Between Anatomic and Non-Anatomic Femoral Fixation—Systematic Review
Source: Children (Basel). 2024 Oct 22;11(11):1275. doi: 10.3390/children11111275 (PMC11592832; doi:10.3390/children11111275)
Supplement: Supplementary file 1 [file children-11-01275-s001.zip › children-3242983-supplementary.pdf]

Risk of bias domains

Study	D1	D2	D3	D4	D5	D6	D7	Overall
Nelitz et al., 2012[8]	+	+	+	+	+	X	+	X
Lind et al., 2014[16]	+	+	+	+	+	X	+	X
Machado et al., 2017[17]	+	+	+	+	+	-	+	-
Nelitz et al.,2017[18]	+	+	+	+	+	-	+	-
Pesenti et al.,2017[19]	+	+	+	+	+	X	+	X
Uppstrom et al.,2019[20]	+	+	+	+	+	X	+	X
Quinlan et al.,2021[21]	+	+	+	+	-	X	+	X
Schlumberger et al.,2021[22]	+	+	+	+	+	X	+	X
Wang et al., 2023[23]	+	+	+	+	+	X	+	X
Zhang et al.,2023[24]	+	+	+	+	+	X	+	X
Leite et al.,2023[25]	+	+	+	+	-	X	+	X

Domains:
D1: Bias due to confounding.
D2: Bias due to selection of participants.
D3: Bias in classification of interventions.
D4: Bias due to deviations from intended interventions.
D5: Bias due to missing data.
D6: Bias in measurement of outcomes.
D7: Bias in selection of the reported result.

Judgement
X Serious
- Moderate
+ Low
